# Effect of coexisting diabetes mellitus and chronic kidney disease on mortality of cirrhotic patients with esophageal variceal bleeding

**DOI:** 10.1186/s12876-016-0434-3

**Published:** 2016-02-29

**Authors:** Chia-Chi Lung, Zhi-Hong Jian, Jing-Yang Huang, Oswald Ndi Nfor

**Affiliations:** Department of Public Health and Institute of Public Health, Chung Shan Medical University, Taichung City, Taiwan; Department of Family and Community Medicine, Chung Shan Medical University Hospital, Taichung City, Taiwan

**Keywords:** Chronic kidney disease, Cirrhosis, Diabetes mellitus, Esophageal variceal bleeding

## Abstract

**Background:**

Esophageal variceal bleeding (EVB) is a serious and common complication of cirrhosis. Diabetes mellitus (DM) and chronic kidney disease (CKD) increase mortality in patients with cirrhosis. However, whether coexisting DM and CKD increase mortality in cirrhotic patients with EVB remains unclear.

**Methods:**

We enrolled cirrhotic patients hospitalized with the first presentation of EVB from 2005 through 2010 using Longitudinal Health Insurance Database 2005. The hazard ratios (HRs) of 42-day and one-year EVB mortality were calculated using Cox regression model.

**Results:**

We identified 888 patients hospitalized with the first presentation of EVB. Among the cirrhotic patients with EVB, all-cause mortality at 42-day and one-year were 21.3 and 45.0 %, respectively. The respective HRs for the 42-day and one-year mortality were 1.80 (95 % confidence interval [CI], 1.10–2.97) and 1.52 (95 % CI, 1.06–2.17) for patients with CKD and 0.79 (95 % CI, 0.57–1.10) and 0.88 (95 % CI, 0.71–1.09) for patients with DM. Specifically, coexisting CKD and DM increased the 42-day and one-year mortality with respective HRs of 1.99 (95%CI, 1.03–3.84) and 1.84 (95%CI, 1.14–2.98) compared with those without CKD and DM. The HRs for 42-day and 1-year mortality in female patients with DM and CKD were 4.03 (95%CI, 1.40–11.59) and 2.84 (95%CI, 1.31–6.14) respectively, and were 2.93 (95%CI, 1.14–7.57) and 2.42 (95%CI, 1.28–4.57) in male patients with DM and CKD.

**Conclusion:**

We identified that coexisting DM and CKD increased risk of mortality at 42 days and 1 year following EVB.

## Background

Bleeding from esophageal varices is a life-threatening condition with an annual mortality of 57 %. Nearly half these deaths occur within 6 weeks from the initial episode of bleeding [1]. Various factors have been proposed as predictors of outcome of variceal bleeding, some of which include age, gender, stage of cirrhosis, etiology, and associated conditions like renal failure and diabetes mellitus (DM) [2–5].

Renal function is a critical prognostic factor in cirrhotic patients with esophageal variceal bleeding (EVB) [6, 7]. Patients with chronic kidney disease (CKD) have many long-term complications, such as increased immunocompromised status [8], as well as an increased risk of metabolic disorders [9], and cardiovascular events [10]. Physiological mechanisms contributing to an increased bleeding tendency included uremic platelet dysfunction, use of antiplatelet agents, and anticoagulants [11, 12]. In addition, usage of aspirin is associated with the occurrence of EVB in cirrhotic patients [13].

The Verona Diabetes Study, a population-based study with 7,148 patients with DM, showed an increased risk of mortality from chronic liver disease and cirrhosis compared with the general population [14]. Insulin resistance, a characteristic feature of DM, has been proven to be a predictor of portal hypertension [15] and the development of esophageal varices [16]. In a hospital-based study with 146 patients with cirrhosis, DM significantly correlated with gastroesophageal variceal bleeding [2].

The presence of metabolic syndrome and the number of metabolic syndrome components have been associated with higher prevalence of CKD [9]. CKD is one of the most common long-term complications of DM [17]. The prevalence of DM and CKD in Taiwan has been reported as 7,570 and 892 per 100,000 population, respectively [18, 19]. How DM and/or CKD per se, is/are independent mortality risk factors, and how they further increase the risk of mortality is still unclear. This study aimed to investigate DM and CKD on mortality of cirrhotic patients with first presentation of EVB.

## Methods

### Data source

This retrospective cohort study used data from the Longitudinal Health Insurance Database 2005 (LHID2005), which is a subset of the National Health Insurance Research Database (NHIRD). The LHID2005 database was derived by the Bureau of National Health Insurance, Ministry of Health and Welfare of Taiwan and maintained by the National Health Research Institutes so as to make it accessible for research purposes. The LHID2005 is broadly used in academic studies [20–23]. LHID2005 contains all the original claims data of one million out of 23 million National Health Insurance enrollees, randomly sampled from the year 2005 registry for beneficiaries of the NHIRD. There was no significant difference in the age and sex distribution between patients in the LHID2005 and the original NHIRD [24]. The use of the data was reviewed and granted by the National Health Research Institutes. The source data was encrypted and the data extracted was anonymous. This study was approved by the Institutional Review Board of the Chung-Shan Medical University Hospital, Taiwan.

### Patient identification

This retrospective study included cirrhotic patients who were hospitalized with a first presentation of EVB between 2005 and 2010. Subjects with incomplete information, such as sex and registry data were excluded. EVB was confirmed by the International Classification of Diseases, Ninth Revision, Clinical Modification (ICD-9-CM) code (ICD-9-CM code 456.0 and 456.20) and esophageal variceal ligation or sclerotherapy.

### Variables of exposure

To reduce bias, the diagnoses of comorbidities were confirmed by more than two outpatient visits or one admission in 1 year. Comorbidities were defined using the following ICD-9-CM codes: DM (250), CKD (585 and 586), hepatitis B virus (HBV) infection (070.2, 070.3, and V02.61), hepatitis C virus (HCV) infection (070.41, 070.44, 070.51, 070.54, 070.7, and V02.62), alcohol-related disorders (291, 303, 305.00–305.03, and 571.0–571.3), hepatocellular carcinoma (HCC) (155.0 and 155.2), ascites (789.5 or ICD-9 Volume 3 procedure code 54.91), hepatic encephalopathy (572.2), spontaneous bacterial peritonitis (SBP) (567.2, 567.8, or 567.9, excluding the procedure codes for the abdominal surgery), chronic obstructive pulmonary disease (COPD) (490, 491, 492, 494, and 496), asthma (493), pulmonary tuberculosis (TB) (010 - 012), acute coronary syndrome (410–414)), cerebrovascular accident (430–438), and bacterial infections. The bacterial infections during hospitalization included pneumonia (ICD-9-CM 481–487, excluding 484), liver abscess (ICD-9-CM 572.0), empyema (ICD-9-CM 510), cellulitis (ICD-9-CM 681 and 682), necrotizing fasciitis (ICD-9-CM 728.86), central nervous system infection (ICD-9-CM 324 and 320), sepsis (ICD-9-CM 038 and 790.7), infective endocarditis (ICD-9-CM 421), urinary tract infection (ICD-9-CM 590.1, 595.0, 595.9, and 599.0), biliary tract infection (ICD-9-CM 574.00, 574.01, 574.30, 574.31, 574.60, 574.61, 574.80, 574.81, 575.0, and 576.1), septic arthritis (ICD-9-CM 711), and perianal abscess (ICD-9-CM 566).

### Statistical analysis

All analyses were made using SAS 9.3 software (SAS Institute, Cary, NC). Chi square test was used to exam the differences in sociodemographic characteristics and comorbidities. Multivariate Cox proportional hazards regression was performed to determine mortality for independent variables, such as sex, age, low income, urbanization, comorbidities, etiology, and complications of cirrhosis. Furthermore, in order to evaluate the effect of coexisting DM and CKD on all-cause mortality, 4 separate models were conducted: 42-day mortality of all patients (Model 1), 42-day mortality stratified by gender (Model 2), 1-year mortality of all patients (Model 3), and 1-year mortality stratified by gender (Model 4). All comparisons with a *p*-value < 0.05 were considered statistically significant.

## Results

The demographic characteristics and comorbidities of cirrhotic patients with EVB are listed in Table 1. The 42-day and 1-year EVB mortalities were 21.3 and 45.0 %, respectively. HBV, CKD, HCC, and hospitalization due to ascites, hepatic encephalopathy, and SBP were more common in non-survivors. The possible etiologies of CKD were as follows: diabetes alone, 3 cases (4.6 %), diabetes + hypertension, 8 (12.3 %), diabetes + hypertension + coronary artery disease, 2 (3.1 %), diabetes + hypertension + hyperlipidemia, 2 (3.1 %), diabetes + hypertension + coronary artery disease + hyperlipidemia, 5 (7.7 %), diabetes + other etiologies, 4 (6.1 %), and etiologies other than diabetes, 41 (63.1 %). For CKD patients with or without DM, the 42-day mortalities were 41.7 % (10/24) and 29.3 % (12/41), and the 1-year mortality were 79.2 % (19/24) and 56.1 % (23/41), respectively (data not shown).

**Table 1:** Characteristics of cirrhotic patients with first esophageal variceal bleeding and mortality, Taiwan, 2005–2010

	42-day mortality			One-year mortality		
	Survivors (*n* = 699) (%)	Death (*n* = 189) (%)	*p*-value	Survivors (*n* = 488) (%)	Death (*n* = 400) (%)	*p*-value
Year of first diagnosis of EVB						
2005 (*n* = 167)	132 (18.9)	35 (18.5)	0.909	84 (17.2)	83 (20.8)	0.585
2006 (*n* = 151)	122 (17.4)	29 (15.3)	0.808	84 (17.2)	67 (16.8)	0.864
2007 (*n* = 142)	101 (14.4)	41 (21.7)	0.170	72 (14.8)	70 (17.5)	0.686
2008 (*n* = 134)	102 (14.6)	32 (16.9)	0.769	79 (16.2)	55 (13.7)	0.729
2009 (*n* = 146)	120 (17.2)	26 (13.8)	0.648	83 (17.0)	63 (15.7)	0.864
2010 (*n* = 148)	122 (17.5)	26 (13.8)	0.615	86 (17.6)	62 (15.5)	0.793
Sex						
Female (*n* = 243)	202 (28.9)	41 (21.7)	0.049	151 (30.9)	92 (23.0)	0.008
Age at the first diagnosis of EVB						
< 50 (*n* = 261)	212 (30.3)	49 (25.9)	0.433	165 (33.8)	96 (24.0)	0.002
50–69 (*n* = 437)	335 (47.9)	102 (54.0)	0.302	230 (47.1)	207 (51.8)	0.171
≥70 (*n* = 190)	152 (21.8)	38 (20.1)	0.626	93 (19.1)	97 (24.2)	0.061
Complication of cirrhosis						
HCC (*n* = 359)	247 (35.3)	112 (59.3)	<0.001	127 (26.0)	232 (58.0)	<0.001
Infection during hospitalization (*n* = 58)	40 (5.7)	18 (9.5)	0.061	26 (5.3)	32 (8.0)	0.109
Previous episodes of decompensation required hospitalization within 1 year before EVB						
Ascites						
0 (*n* = 711)	577 (82.6)	134 (70.9)	<0.001	417 (85.4)	294 (73.5)	<0.001
1 (*n* = 114)	82 (11.7)	32 (16.9)	0.058	52 (10.7)	62 (15.5)	0.038
≥ 2 (*n* = 63)	40 (5.7)	23 (12.2)	0.002	19 (3.9)	44 (11.0)	<0.001
Hepatic encephalopathy						
0 (*n* = 789)	628 (89.9)	161 (85.2)	0.247	447 (91.6)	342 (85.4)	0.026
1 (*n* = 60)	47 (6.7)	13 (6.9)	0.940	31 (6.4)	29 (7.3)	0.724
≥ 2 (*n* = 39)	24 (3.4)	15 (7.9)	0.041	10 (2.0)	29 (7.3)	0.001
SBP						
0 (*n* = 846)	670 (95.9)	176 (93.1)	0.329	477 (97.8)	369 (92.3)	<0.001
1 (*n* = 35)	28 (4.0)	7 (3.7)	0.850	10 (2.0)	25 (6.2)	0.003
≥ 2 (*n* = 7)	1 (0.1)	6 (3.2)	<0.001	1 (0.2)	6 (1.5)	0.028
Etiology of cirrhosis						
HBV (*n* = 340)	250 (35.8)	90 (47.6)	0.003	168 (34.4)	172 (43.0)	0.009
HCV (*n* = 306)	248 (35.5)	58 (30.7)	0.219	168 (34.4)	138 (34.5)	0.982
Alcoholism (*n* = 382)	313 (44.8)	69 (36.5)	0.042	236 (48.4)	146 (36.5)	<0.001
Comorbidities						
DM (*n* = 332)	270 (38.6)	62 (32.8)	0.142	187 (38.3)	145 (36.3)	0.526
CKD (*n* = 65)	43 (6.2)	22 (11.6)	0.010	23 (4.7)	42 (10.5)	0.001
COPD (*n* = 216)	174 (24.9)	42 (22.2)	0.448	124 (25.4)	92 (23.0)	0.405
Asthma (*n* = 87)	69 (9.9)	18 (9.5)	0.887	49 (10.0)	38 (9.5)	0.787
TB (*n* = 28)	24 (3.4)	4 (2.1)	0.358	18 (3.7)	10 (2.5)	0.313
Previous episode of acute coronary syndrome (*n* = 184)	145 (20.7)	39 (20.6)	0.974	96 (19.7)	88 (22.0)	0.395
Previous episode of cerebrovascular accident (*n* = 102)	73 (10.4)	29 (15.3)	0.061	45 (9.2)	57 (14.3)	0.019
Low income						
Yes (*n* = 24)	20 (2.9)	4 (2.1)	0.575	7 (1.4)	17 (4.3)	0.010
Urbanization						
Urban (*n* = 438)	339 (48.5)	99 (52.4)	0.357	246 (50.4)	192 (48.0)	0.475
Normal (*n* = 316)	243 (34.8)	73 (38.6)	0.357	160 (32.8)	156 (39.0)	0.120
Rural (*n* = 134)	117 (16.7)	17 (9.0)	0.030	82 (16.8)	52 (13.0)	0.214

*CKD* chronic kidney disease, *COPD* chronic obstructive pulmonary disease, *DM* diabetes mellitus, *EVB* esophageal variceal bleeding, *HBV* hepatitis B virus, *HCC* hepatocellular carcinoma, *HCV* hepatitis C virus, *SBP* spontaneous bacterial peritonitis, *TB* pulmonary tuberculosis

Table 2 shows the HRs for 42-day and 1-year mortality. At 42 days following EVB, the risk of mortality was high in patients with CKD (hazard ratio [HR], 1.80; 95 % confidence interval [CI], 1.10–2.97], HCC (HR, 2.13; 95 % CI, 1.54–2.95), and previous hospitalization due to ascites and SBP. Similarly, at 1 year following EVB, risk of mortality was also high in men (HR,1.54; 95 % CI, 1.19–2.00), CKD (HR,1.52; 95 % CI, 1.06–2.17), HCC (HR, 2.48; 95%CI, 1.99–3.10), infections during hospitalization (HR:1.50; 95 % CI, 1.03–2.18), and previous hospitalization due to ascites and SBP.

**Table 2:** Estimation of hazard ratios of mortality in patients with esophageal variceal bleeding between 2005 and 2010 using cox proportional model

	42-day mortality			One-year mortality		
	HR	95 % C.I.	*p*-value	HR	95 % C.I.	*p*-value
Sex (reference: Female)						
Male	1.37	0.93–2.02	0.109	1.54	1.19–2.00	0.001
Age at the first diagnosis of EVB (reference: <50)						
50–69	0.93	0.63–1.38	0.721	1.03	0.77–1.38	0.831
≥ 70	0.77	0.45–1.34	0.361	1.24	0.85–1.81	0.269
Comorbidities						
DM	0.79	0.57–1.10	0.163	0.88	0.71–1.09	0.241
CKD	1.80	1.10–2.97	0.021	1.52	1.06–2.17	0.023
COPD	0.88	0.59–1.31	0.533	0.85	0.65–1.12	0.251
Asthma	1.19	0.70–2.00	0.526	1.08	0.75–1.55	0.699
TB	0.68	0.27–1.88	0.460	0.65	0.34–1.24	0.191
Previous episode of acute coronary syndrome	1.01	0.68–1.51	0.954	1.04	0.79–1.36	0.779
Previous episode of cerebrovascular accident	1.54	0.99–2.38	0.054	1.21	0.89–1.65	0.214
Etiology of cirrhosis						
HBV	1.13	0.82–1.54	0.465	1.07	0.86–1.33	0.559
HCV	0.81	0.57–1.14	0.224	0.86	0.68–1.09	0.217
Alcoholism	0.76	0.52–1.11	0.157	0.74	0.57–0.97	0.031
Complication of cirrhosis						
HCC	2.13	1.54–2.95	<0.001	2.48	1.99–3.10	<0.001
Infection during hospitalization	1.65	0.99–2.75	0.055	1.50	1.03–2.18	0.035
Previous episodes of decompensation required hospitalization within 1 year before EVB						
Ascites (reference: 0)						
1	1.66	1.10–2.52	0.017	1.60	1.18–2.18	0.003
≥ 2	1.77	1.04–3.02	0.036	1.73	1.18–2.55	0.005
Hepatic encephalopathy (reference: 0)						
1	0.98	0.54–1.79	0.949	1.09	0.72–1.67	0.680
≥ 2	1.09	0.59–1.99	0.793	1.28	0.82–1.99	0.271
SBP (reference: 0)						
1	0.63	0.28–1.41	0.258	1.20	0.76–1.92	0.435
≥ 2	3.42	1.38–8.50	0.008	2.93	1.24–6.93	0.015
Low income	0.70	0.25–1.99	0.503	1.63	0.95–2.79	0.078
Urbanization (reference: Urban)						
Sub-urban	1.02	0.75–1.39	0.891	1.09	0.87–1.35	0.455
Rural	0.58	0.34–0.98	0.043	0.84	0.61–1.15	0.270

*CI* confidence interval, *CKD* chronic kidney disease, *COPD* chronic obstructive pulmonary disease, *DM* diabetes mellitus, *EVB* esophageal variceal bleeding, *HBV* hepatitis B virus, *HCC* hepatocellular carcinoma, *HCV* hepatitis C virus, *HR* hazard ratio, *SBP* spontaneous bacterial peritonitis, *TB* pulmonary tuberculosis

Table 3 illustrates the adjusted HRs of EVB mortality at 42 days and 1 year in patients with either DM, CKD, or both by gender. For 42-day mortality (Model 1), the HRs were 1.99 (95 % CI, 1.03–3.84) and 0.72 (95%CI, 0.50–1.02) among DM patients with or without CKD, respectively. The HR for patients with CKD was 1.25 (95 % CI, 0.62–2.52). When stratified by gender and disease combinations (Model 2), the HRs were higher in female diabetic patients with CKD (HR, 4.03; 95 % CI, 1.40–11.59) and male diabetic patients with CKD (HR, 2.93; 95 % CI, 1.14–7.57).

**Table 3:** Adjusted Risk for 42-Day and One-Year Mortality Stratified by DM, Chronic Kidney Diseases, and Sex

	42-day mortality						One-year mortality					
	Model 1^a^			Model 2^b^			Model 3^a^			Model 4^b^		
	HR	95 % CI	*P* value	HR	95 % CI	*P* value	HR	95 % CI	*P* value	HR	95 % CI	*P* value
All patients												
Non-DM & CKD	Ref.						Ref.					
Only DM	0.72	0.50–1.02	0.062				0.80	0.64–1.02	0.066			
Only CKD	1.25	0.62–2.52	0.541				1.07	0.65–1.76	0.801			
DM + CKD	1.99	1.03–3.84	0.042				1.84	1.14–2.98	0.012			
P for DM × CKD interaction	0.100							0.028				
Women												
Non-DM & CKD				Ref.						Ref.		
Only DM				1.11	0.56–2.21	0.772				0.77	0.49–1.21	0.260
Only CKD				3.40	0.75–15.48	0.113				1.30	0.39–4.28	0.672
DM + CKD				4.03	1.40–11.59	0.010				2.84	1.31–6.14	0.008
Men												
Non-DM & CKD				2.00	1.14–3.52	0.016				1.61	1.13–2.29	0.009
Only DM				1.25	0.67–2.32	0.487				1.32	0.90–1.92	0.154
Only CKD				2.02	0.83–4.94	0.123				1.64	0.90–2.98	0.108
DM + CKD				2.93	1.14–7.57	0.026				2.42	1.28–4.57	0.007
P for gender × diseases interaction				0.920						0.559		

*CI* confidence interval, *CKD* chronic kidney disease, *DM* diabetes mellitus, *HR* hazard ratio, *Ref* reference

^a^Adjusted for sex, age, comorbidities, etiology of cirrhosis, complications of cirrhosis, low income and urbanization

^b^Adjusted for age, comorbidities, etiology of cirrhosis, complications of cirrhosis, low income and urbanization

For 1-year mortality, the HRs were 1.84 (95 % CI, 1.14–2.98) and 0.80 (95 % CI, 0.64–1.02) in diabetic patients with or without CKD (Model 3). The HR for patients with CKD was 1.07 (95 % CI, 0.65–1.76). There was significant interaction between DM and CKD (*p* = 0.028). In Model 4, the HRs for male and female diabetic patients with CKD were 2.84 (95 % CI, 1.31–6.14) and 2.42 (95 % CI, 1.28–4.57), respectively.

## Discussion

We found that coexistence of CKD and DM was independently associated with 42-day and 1-year mortality in both sexes. These risk factors are easy to identify following the initial EVB event and are valuable for predicting clinical outcomes. They may also be useful for guiding the clinical management of cirrhotic patients with EVB. Identifying patients at high risk will be important for cost-effective management of EVB.

Globally, 57 % of cirrhosis is attributable to either hepatitis B (30 %) or hepatitis C (27 %) [25]. Alcohol consumption is another important cause, accounting for about 20 % of the cases. The seroprevalence of HBV and HCV was 17.3 and 4.4 % in Taiwan, respectively [26]. More than 70 % of cirrhosis and HCC were the sequelae of chronic HBV infection in 1990s [27]. Our results showed that HBV infection was higher in non-survivors and the prevalence of HBV infection in HCC was 46.2 %. When HBV infection was put into the multivariate model for analyses, there was no significant association between HBV infection and EVB mortality. The efficacy of universal immunization has been proven with substantial reductions of HBV carriage in children, adolescents and young adults since 1984 [28]. HCC in Taiwan also falls after universal hepatitis B vaccination [29]. We also showed improvement in the 42-day mortality rate over time perhaps due to treatment advances, such as variceal ligation, appropriate vasoconstrictor usage, and antiviral treatment for the underlying cirrhosis [30, 31].

Bleeding from ruptured esophageal varices is the most severe complication of cirrhosis and 6-week mortality rates have been reported to be 15–20 % [30]. The inpatient bleeding rate among cirrhotic patients has been reported as 13 % [32]. Around 30 to 60 % of cirrhotic patients suffer from DM with insulin resistance and hyperinsulinemia [33]. The presence of DM appears to be associated with failure to control esophageal variceal bleeding and re-bleeding [34]. Hyperglycemia induces splanchnic hyperemia, increases portal pressure and azygos vein blood flow, and may increase the risk of variceal bleeding [35, 36]. However, in this study, DM individually failed to show significant association with 42-day and 1-year mortality.

In an analysis involving 2,592 cirrhotic patients hospitalized with SBP in 2004, the respective HRs for 30-day and 1-year mortality were 1.37 (95%CI, 0.85–2.21) and 1.37 (95%CI, 1.01–1.84) in patients with CKD [37]. Increased long-term mortality rates of SBP in cirrhotic patients may be attributed to the impaired immune functions caused by CKD [38]. Hung et al. evaluated 4,932 cirrhotic patients with hepatic encephalopathy and showed that the adjusted HR of 3-year mortality for CKD was 1.93 (95 % CI, 1.55–2.40) compared with those with normal renal function [39]. However, patients with end-stage renal disease (ESRD) receiving hemodialysis had better 3 year survival rate (HR, 0.66; 95 % CI, 0.46–0.94) than those with CKD. This implied that CKD may be associated with poor clearance of circulatory neurotoxic substances that increases the susceptibility to mortality in cirrhotic patients with hepatic encephalopathy. Hung et al. evaluated 6,740 cirrhotic patients who were hospitalized with EVB in 2007 and showed that ESRD was associated with 1-year mortality (HR,1.50; 95 % CI, 1.18–1.91), but not a risk factor for 42-day mortality (HR,1.19; 95 % CI, 0.79–1.78) [5]. They implicated that the ESRD-related platelet dysfunction contributed higher EVB and mortality. In our study, we identified that CKD was independent prognostic factor for both 42-day and one-year mortality in cirrhotic patients with first presentation of EVB.

Coexisting DM and CKD are important prognostic factors in cirrhotic patients regardless of the causes of liver diseases. CKD and DM have many long-term complications such as increased immunocompromised status, as well as increased risk of metabolic disorders and cardiovascular events [40–42]. DM increases portal blood flow secondary to fluctuating blood sugar levels leading to an increase in portal pressure [35, 43]. There is increased bleeding tendency due to uremic platelet dysfunction, use of antiplatelet agents, and anticoagulants [11, 12]. In addition, usage of aspirin increases the occurrence of EVB in cirrhotic patients [13]. These complications could indicate why the 42-day and 1-year overall mortality was higher among the coexisting DM and CKD than DM or CKD individuals. A gender-stratified comparative analysis indicated that coexisting DM and CKD exhibited mortality risk in both genders.

In a cohort study with patients with chronic hepatitis C (CHC), patients with new-onset diabetes subsequently were found to have an increased risk of developing cirrhosis, or decompensation in those with established cirrhosis [44]. Persico et al. retrospectively evaluated 852 consecutive patients (726 CHC and 126 chronic hepatitis B) who had undergone liver biopsy [45]. Liver fibrosis (odds ratio [OR], 4.70; 95%CI, 2.75–8.03) was independent risk factors for the presence of significant steatosis (>30 %) in patient with CHC. Camma et al. analyzed 104 patients with CHC cirrhosis (Child-Pugh class A) receiving upper gastrointestinal endoscopy [16]. They found a high homeostasis model assessment score (OR, 1.37; 95 % CI, 1.01–1.86) as an independent predictor of the presence of esophageal varices. Cirrhosis, per se, independently by the viral etiology, may be associated with the development of insulin resistance and diabetes.

Although HCV and HBV infection were not associated with increased risk of mortality, they have been reported to be associated with renal diseases. HCV is also associated with extra-hepatic diseases, including various types of glomerulonephritis, even in the absence of cirrhosis [46]. A high baseline HCV viral load was an independent predictor of CKD [47]. Soma et al. indicated that HCV infection leads to a rapid decline in the renal function of patients with diabetic nephropathy [48]. HBV-related renal injury is associated with the deposition of immune complexes of HBV antigens and host antibodies [49]. Untreated chronic HBV infection is also associated with increased risk of CKD [50].

Hepatic steatosis or fatty liver is characterized by lipid accumulation within the cytoplasm of hepatocytes, and includes a spectrum of liver disease from a benign simple steatosis, steatohepatitis, to fibrosis [51]. In Taiwan, the prevalence of non-alcoholic fatty liver disease (NAFLD) is about 11.5 % [52], and the rates are higher in subgroups, from 66.4 % in healthy taxi drivers [53] to 80 % in obese individuals enrolled in weight reduction programs [54]. In a cross-sectional community study that included 11.4 % (372/3,260) individuals with elevated alanine aminotransferase (ALT), NAFLD was the most common cause of ALT elevation with a prevalence of 33.6 %, and followed by HBV (28.5 %), unexplained cause (21.8 %), HCV (13.2 %), both HBV and HCV (2.2 %), and excess alcohol consumption (0.8 %) [55]. Approximately 1 % of patients with NAFLD develop cirrhosis [56], where as in non-alcoholic steatohepatitis (NASH), the estimated figure is up to 20 % [57]. However, NAFLD was not available in the NHIRD.

Our data included all EVB patients hospitalized in a variety of hospitals in Taiwan; hence, selection bias was minimized. There were some significant limitations to this study. First, the basic laboratory data (e.g. prothrombin times, bilirubin, creatinine, and albumin levels) were not available. For the severity of cirrhosis, Child-Pugh or Model of End-Stage Liver Disease (MELD) scores could not be calculated using the ICD-9 coding in the database. We put previous episodes of decompensation that required hospitalization, such as asictes, hepatic encephalopathy, and SBP into multiple variable analyses. This information could obviate the important limitation of not having laboratory data to calculate the MELD score. Second, the exact cases of NAFLD-related cirrhosis were not available. Data on NAFLD-related cirrhosis are limited and only one case report has been reported in Taiwan [58]. Third, the diagnoses of EVB and other comorbidities were based on ICD-9 codes, and misclassification could be possible. We included cirrhotic patients who were hospitalized with first presentation of EVB and received esophageal variceal ligation or sclerotherapy to minimize ascertainment bias.

## Conclusions

This study provides evidence that coexistence of CKD and DM has a higher impact on 42-day and one-year mortality than DM or CKD individually.

## Abbreviations

ALT: alanine aminotransferase
CHC: chronic hepatitis C
CI: confidence interval
CKD: chronic kidney disease
COPD: chronic obstructive pulmonary disease
DM: diabetes mellitus
ESRD: end-stage renal disease
EVB: esophageal variceal bleeding
HBV: hepatitis B virus
HCC: hepatocellular carcinoma
HCV: hepatitis C virus
HR: hazard ratio
ICD-9-CM: International Classification of Diseases, Ninth Revision, Clinical Modification
LHID2005: Longitudinal Health Insurance Database 2005
MELD: Model of End-Stage Liver Disease
NAFLD: non-alcoholic fatty liver disease
NASH: non-alcoholic steatohepatitis
NHIRD: National Health Insurance Research Database
OR: odds ratio
SBP: spontaneous bacterial peritonitis
TB: pulmonary tuberculosis
